# Ginsenoside RK1 Induces Ferroptosis in Hepatocellular Carcinoma Cells through an FSP1-Dependent Pathway

**DOI:** 10.3390/ph17070871

**Published:** 2024-07-02

**Authors:** Yulang Jiang, Yongxin Yu, Ziyang Pan, Ziyuan Wang, Mingyu Sun

**Affiliations:** 1 Shuguang Hospital Affiliated to Shanghai University of Traditional Chinese Medicine, Shanghai 201203, China; albert_jylss@163.com (Y.J.); yongxin0705@163.com (Y.Y.); ashleytzuyang@163.com (Z.P.); 2 Internal Medicine in Chinese Medicine, Shanghai University of Traditional Chinese Medicine, Shanghai 201203, China; 3 Key Laboratory of Liver and Kidney Diseases, Institute of Liver Diseases, Shuguang Hospital Affiliated to Shanghai University of Traditional Chinese Medicine, Shanghai 201203, China

**Keywords:** ginsenoside RK1, FSP1, antioxidant, ferroptosis, HCC

## Abstract

Background: Hepatocellular carcinoma (HCC), currently ranking as the third most lethal malignancy, poses a grave threat to human health. Ferroptosis, a form of programmed cell demise, has emerged as a promising therapeutic target in HCC treatment. In this study, we investigated the impact of ginsenoside RK1 on ferroptosis induction in HCC cells and elucidated the underlying mechanisms. Methods: The HCC cell line HepG2 was utilized to evaluate the effects of ginsenoside RK1. Distinct dosages of ginsenoside RK1 (25 μM, 50 μM, and 100 μM) were selected based on half-maximal inhibitory concentration (IC50) values. Cellular viability was assessed using a CCK8 assay, cytotoxicity was measured via lactate dehydrogenase (LDH) release assay, and colony-forming ability was evaluated using the clone formation assay. Various inhibitors targeting apoptosis (Z-VAD-FMK 20 μM), necrosis (Nec-1, 10 μM), and ferroptosis (Fer-1, 10 μM; Lip-1, 1 μM) were employed to assess ginsenoside RK1’s impact on cell demise. Intracellular levels of key ions, including glutathione (GSH), malondialdehyde (MDA), and iron ions, were quantified, and the protein expression levels of ferroptosis-related genes were evaluated. The sensitivity of HCC cells to ferroptosis induction by ginsenoside RK1 was examined following the overexpression and silencing of the aforementioned target genes. Results: Ginsenoside RK1 exhibited an inhibitory effect on HCC cells with an IC50 value of approximately 20 μM. It attenuated cellular viability and colony-forming capacity in a dose-dependent manner, concurrently reducing intracellular GSH levels and increasing intracellular Malondialdehyde (MDA) and iron ion contents. Importantly, cell demise induced by ginsenoside RK1 was specifically counteracted by ferroptosis inhibitors. Furthermore, the modulation of Ferroptosis suppressor protein 1 (FSP1) expression influenced the ability of ginsenoside RK1 to induce ferroptosis. FSP1 overexpression or silencing enhanced or inhibited ferroptosis induction by ginsenoside RK1, respectively. Conclusions: Ginsenoside RK1 enhances ferroptosis in hepatocellular carcinoma through an FSP1-dependent pathway.

## 1. Introduction

Hepatocellular carcinoma (HCC), a formidable malignancy notorious for its high morbidity and mortality rates, poses a significant threat to human health [1]. Despite surgical resection and liver transplantation standing as the most efficacious interventions, the nonspecific clinical presentation of HCC often leads to late-stage diagnosis [2]. This highlights the critical need for expanded therapeutic strategies. Traditional Chinese medicine (TCM) emerges as a promising avenue, offering multifaceted therapeutic benefits targeting various stages of HCC progression, warranting further comprehensive exploration and research [3,4].

Ginsenoside RK1, a rare compound derived from ginseng via glycosyl removal, exhibits diverse biological activities, including anticancer, antiviral, anti-inflammatory, and antitumor properties. Its demonstrated efficacy in inhibiting tumor proliferation positions it as a compelling candidate for anticancer drug development [5,6,7,8]. Multiple studies have consistently demonstrated the significant inhibitory impact of ginsenoside RK1 on the proliferation of hepatocellular carcinoma cells. Its multifaceted mechanism of action is noteworthy. Ginsenoside RK1 effectively induces cell cycle arrest in the G0/G1 phase, thereby slowing the proliferation rate of these cancer cells [9]. Furthermore, this compound activates both the mitochondrial pathway and the endoplasmic reticulum stress response, initiating the apoptotic cascade in hepatocellular carcinoma cells and ultimately leading to cell death. Moreover, ginsenoside RK1 exhibits the ability to hinder the migration and invasion capabilities of these cells, thus mitigating the potential for metastasis.

Ferroptosis, an iron-ion-dependent programmed cell death pathway characterized by disrupted intracellular oxidative balance, iron overload, and lipid peroxide accumulation, plays a pivotal role in tumorigenesis, tumor immunity, and response to chemotherapy and radiotherapy [10,11,12]. Sorafenib, the inaugural targeted therapy approved for liver cancer treatment, has recently been found to trigger ferroptosis in certain tumor cells. Notably, regulatory proteins involved in ferroptosis also influence the sensitivity of liver cancer to this drug. Furthermore, given that the mechanism of ferroptosis diverges from other forms of programmed cell death, tumor cells originating from specific tissues, in a particular differentiation state, and resistant to conventional chemotherapy, exhibit a remarkable sensitivity to ferroptosis-inducing agents, despite their resistance to apoptosis [13]. Targeting ferroptosis holds significant promise for anticancer drug development [14,15].

Despite ginsenoside RK1’s documented anticancer efficacy in various malignancies, including breast, liver, and lung cancers, its specific mechanism in regulating ferroptosis remains elusive [5,16,17,18]. Our study aims to elucidate the inhibitory effects of ginsenoside RK1 on HCC utilizing HepG2 and Hep3B cell lines. We will assess alterations in ferroptosis sensitivity induced by ginsenoside RK1 through biochemical markers and delineate the underlying mechanisms via RT-qPCR and western blot analysis. This endeavor seeks to unveil ginsenoside RK1’s potential in HCC prevention and treatment, offering novel therapeutic targets and theoretical insights.

## 2. Results

### 2.1. Ginsenoside RK1 Inhibits Hepatocellular Carcinoma Cell Viability by Mediating Ferroptosis 

**Figure 1:**
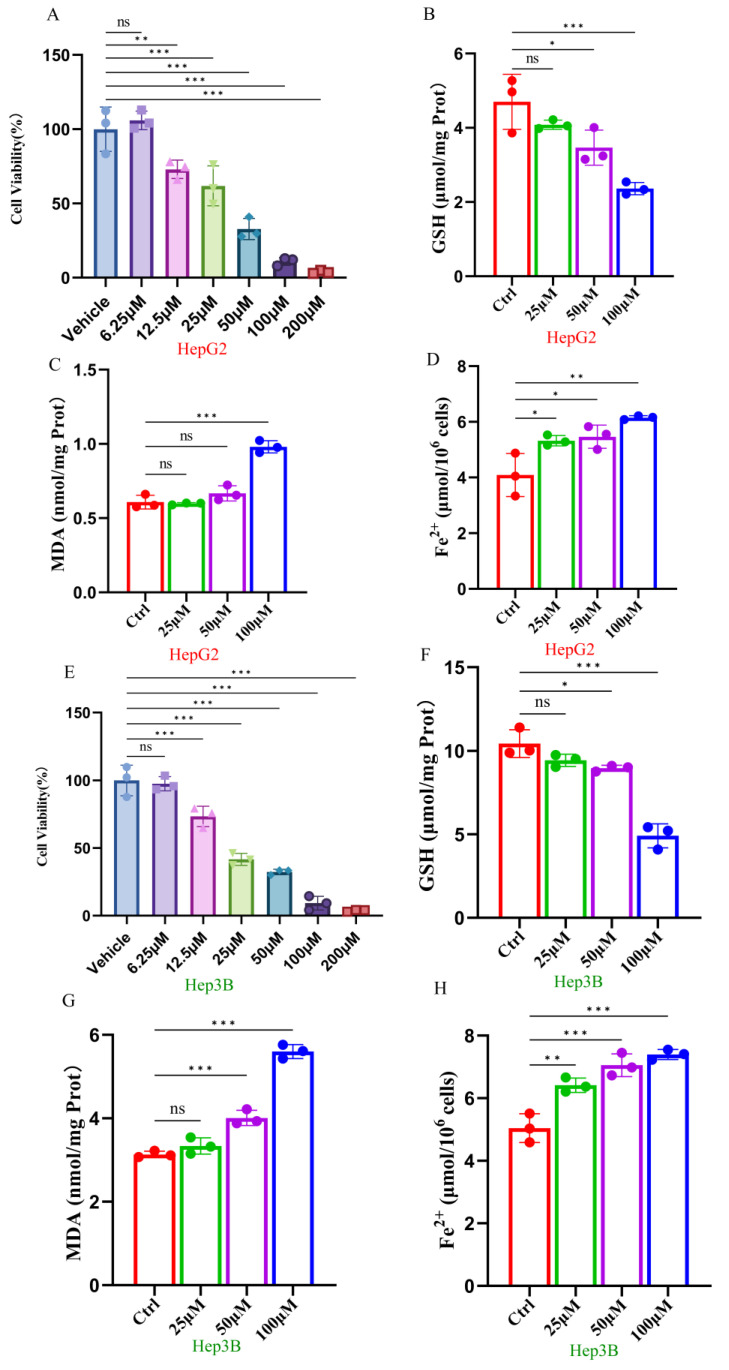
Ginsenoside RK1 induces ferroptosis in hepatocellular carcinoma cells. (**A**) Effects of different concentrations of ginsenoside RK1 on the viability of HepG2 cells (*n* = 3). (**B**) Effects of different concentrations of ginsenoside RK1 on GSH content in HepG2 cells (*n* = 3). (**C**) Effects of different concentrations of ginsenoside RK1 on MDA content in HepG2 cells (*n* = 3). (**D**) Effects of different concentrations of ginsenoside RK1 on iron ion content in HepG2 cells (*n* = 3). (**E**) Effects of different concentrations of ginsenoside RK1 on the viability of Hep3B cells (*n* = 3). (**F**) Effects of different concentrations of ginsenoside RK1 on GSH content in Hep3B cells (*n* = 3). (**G**) Effects of different concentrations of ginsenoside RK1 on MDA content in Hep3B cells (*n* = 3). (**H**) Effects of different concentrations of ginsenoside RK1 on iron ion content in Hep3B cells (*n* = 3). Data are mean ± SEM, *n* ≥ 3; The data between two groups were analyzed by an independent sample *t*-test, and a one-way ANOVA was used to compare the means of three groups, * *p* < 0.05; ** *p* < 0.01; *** *p* < 0.001, ns: no significance.

Compared with the control group, the viability of both HepG2 and Hep3B cells exhibited a significant decline with increasing concentrations of ginsenoside RK1. Notably, a discernible difference in cell viability emerged at concentrations of 25 μM and higher compared to the control group (*p* < 0.01). The IC_50_ values for HepG2 and Hep3B cells were determined to be 41.5 μM and 30.8 μM, respectively (Figure 1A,E). Based on the outcomes of the CCK8 assays, we designated 25 μM as the low-dose group, 50 μM as the medium-dose group, and 100 μM as the high-dose group of ginsenoside RK1 for subsequent investigations. Furthermore, we evaluated alterations in intracellular GSH, MDA, and iron ion levels in hepatocellular carcinoma cells following ginsenoside RK1 stimulation. The findings revealed a dose-dependent reduction in intracellular GSH content in HepG2 cells (Figure 1B) alongside escalated levels of MDA (Figure 1C) and iron ions (Figure 1D). Consistent trends were also observed in Hep3B cells (Figure 1F–H). In summary, these results underscore the inhibitory effects of ginsenosides on hepatocellular carcinoma cell proliferation, implicating ferroptosis as a pivotal mediator in this process.

### 2.2. Effect of Ferroptosis Inhibitors Fer-1 and Lip-1 on the Activity of Ginsenoside RK1

**Figure 2:**
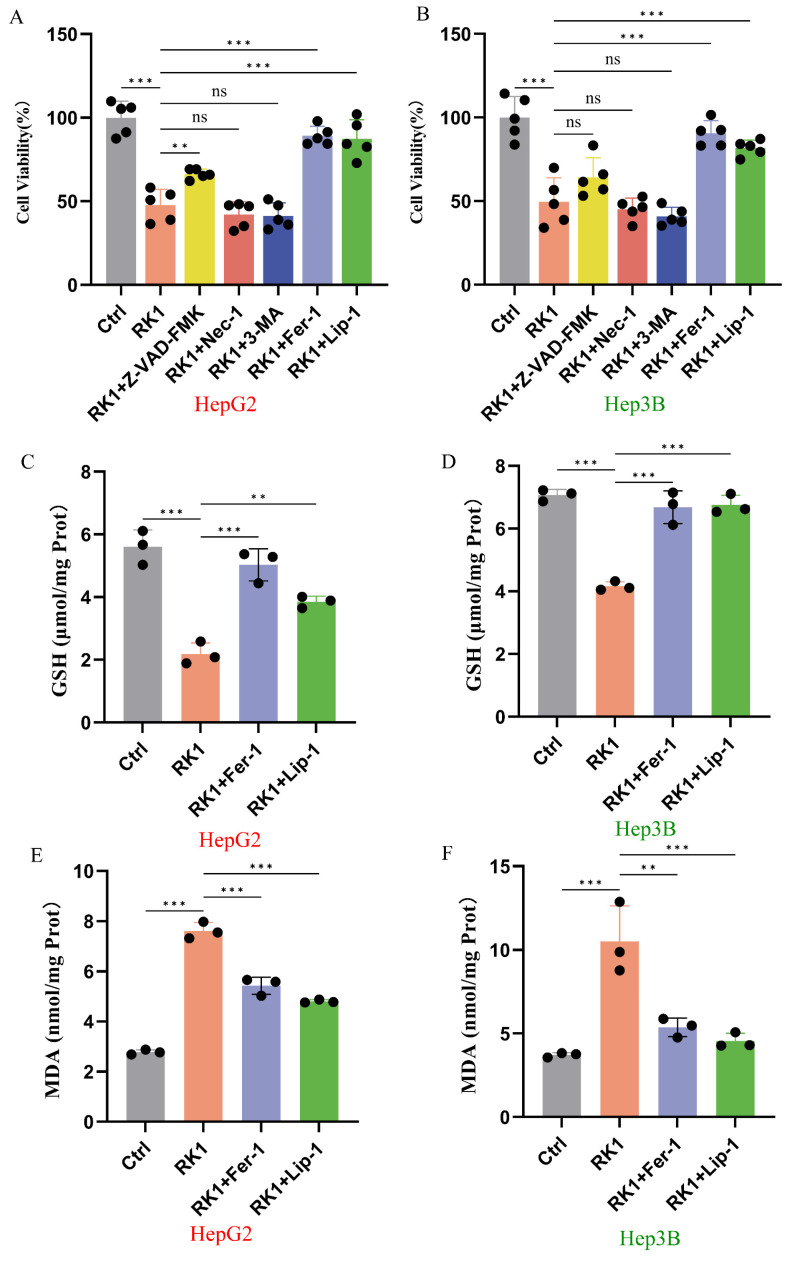
Ferroptosis inhibitors Fer-1 and Lip-1 inhibit ginsenoside RK1-induced ferroptosis. (**A**) Effect of co-stimulation with different cell death inhibitors and ginsenoside RK1 (50 μM) on HepG2 cell viability (*n* = 5). (**B**) Effect of co-stimulation with different cell death inhibitors and ginsenoside RK1 (50 μM) on Hep3B cell viability (*n* = 5). (**C**) Effects of using or not using the ferroptosis inhibitors Fer-1 and Lip-1 on GSH content in HepG2 cells supplemented with ginsenoside RK1 (50 μM) (*n* = 3). (**D**) Effects of using or not using the ferroptosis inhibitors Fer-1 and Lip-1 on GSH content in Hep3B cells supplemented with ginsenoside RK1 (50 μM) (*n* = 3). (**E**) Effects of using or not using the ferroptosis inhibitors Fer-1 and Lip-1 on MDA content in HepG2 cells supplemented with ginsenoside RK1 (50 μM) (*n* = 3). (**F**) Effects of using or not using the ferroptosis inhibitors Fer-1 and Lip-1 on MDA content in Hep3B cells supplemented with ginsenoside RK1 (50 μM) (*n* = 3). Data are mean ± SEM, *n* ≥ 3; data between two groups were analyzed by an independent samples *t*-test, and a one-way ANOVA was used to compare the means of three groups; ** *p* < 0.01; *** *p* < 0.001, ns: no significance.

To delineate the specific modes of hepatocellular carcinoma cell death induced by ginsenoside RK1, we employed various cell death inhibitors to counteract ginsenoside RK1-mediated cell demise. Intriguingly, our investigation revealed that inhibitors targeting apoptosis, necrosis, and autophagy failed to effectively rescue the inhibition of hepatocellular carcinoma cells by ginsenoside RK1 (Figure 2A,B). Subsequent analyses unveiled that the co-administration of Fer-1 and Lip-1, in contrast to the ginsenoside RK1 treatment alone, led to elevated intracellular GSH levels (Figure 2C,D) concurrent with increased intracellular MDA content (Figure 2E,F). This observation suggests that Fer-1 and Lip-1 intervention mitigated the susceptibility to ferroptosis and ameliorated the oxidative stress milieu within hepatocellular carcinoma cells. These findings further corroborate the capacity of ginsenoside RK1 to induce ferroptosis in hepatocellular carcinoma cells. To determine the effect of ginsenoside RK1 on normal hepatocytes, we selected human normal hepatocytes (L02) and mouse normal hepatocytes (AML12) for a cell viability assay using the CCK8 method. The results revealed that ginsenoside RK1 exerted no significant influence on the viability of L02 and AML12 cells at concentrations up to 100 μM (*p* > 0.05). However, at a concentration of 200 μM, ginsenoside RK1 did inhibit the viability of these cells (*p* < 0.05) (refer to Figure S2A,B). For our subsequent experiments, we opted to use ginsenoside RK1 at concentrations of 25 μM, 50 μM, and 100 μM, as these levels did not cause notable damage to healthy hepatocytes.

Furthermore, we investigated the alterations in FSP1 protein and mRNA levels in L02 cells under the influence of ginsenoside RK1. Our findings indicated that varying concentrations of ginsenoside RK1 did not significantly alter the levels of FSP1 in L02 cells (*p* > 0.05) (see Figure S2C–E). This observation suggests an intriguing phenomenon: ginsenoside RK1 appears to specifically target ferroptosis in hepatocellular carcinoma cells, while healthy hepatocytes seem to possess a mechanism that resists ferroptosis induced by ginsenoside RK1. This implies that combining ginsenoside RK1 with ferroptosis inducers could potentially yield a significant synergistic effect, and we intend to explore this topic further in future studies.

Additionally, as an ancient and preserved metabolic mode of cell death, ferroptosis possesses natural resistance genes within cells and tissues. Identifying regulators or checkpoints that influence hypersensitivity to ferroptosis in different cells or tissues will be a focal point of future research in this field.

### 2.3. Ginsenoside RK1 Induces Ferroptosis by Inhibiting the Ferroptosis Defense System

**Figure 3:**
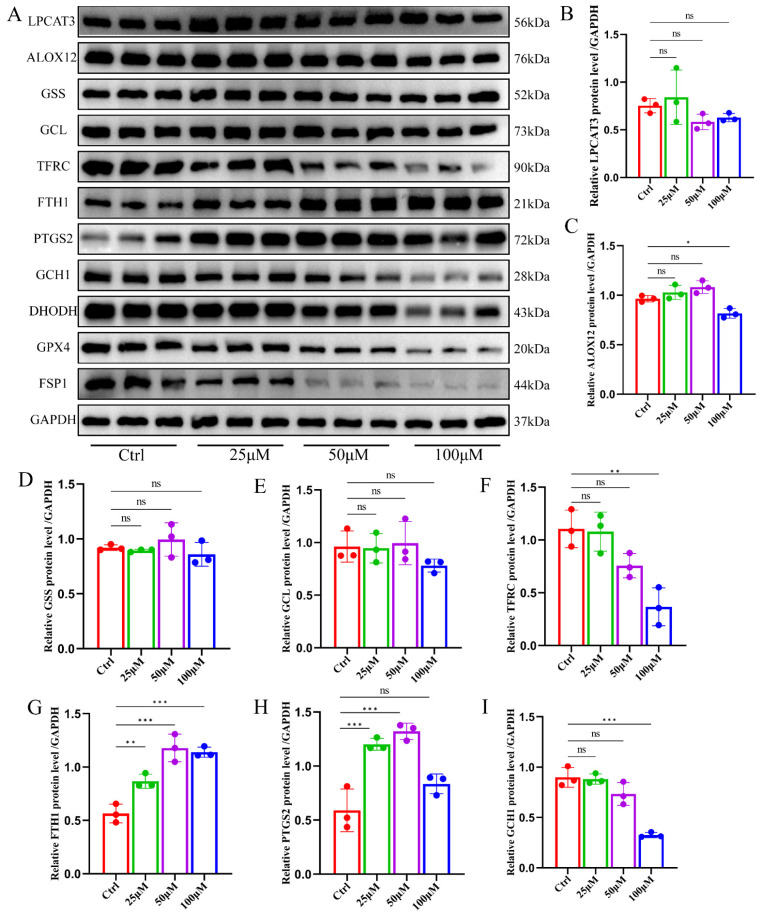

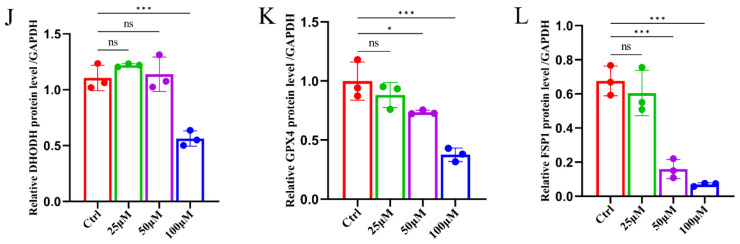
Ginsenoside RK1 promotes ferroptosis by inhibiting ferroptosis defense system activity. (**A**) Representative images of immunoblotting of different concentrations of ginsenoside RK1 on the expression of different proteins in HepG2 cell (*n* = 3). (**B**–**L**) Semi-quantitative statistical plots of protein expression of LPCAT3, ALOX12, GSS, GCL, TFRC, FTH1, PTGS2, GCH1, DHODH, GPX4 and FSP1 in HepG2 cells under the intervention of different concentrations of ginsenoside RK1. Data are mean ± SEM, *n* ≥ 3; data between two groups were analyzed by an independent samples *t*-test, and a one-way ANOVA was used to compare the means of three groups, * *p* < 0.05; ** *p* < 0.01; *** *p* < 0.001, ns: no significance.

Ferroptosis, characterized as a metabolic mode of cell demise, implicates various pathways encompassing iron ion metabolism, fatty acid metabolism, amino acid metabolism, reactive oxygen species metabolism, and energy metabolism, all intricately intertwined. These metabolic processes serve as prerequisites for ferroptosis occurrence, underpinned by inherent cellular defense mechanisms aimed at safeguarding against ferroptosis. Hence, we scrutinized the impact of ginsenoside RK1 intervention on the ferroptosis metabolic landscape in hepatocellular carcinoma cells via Western blot analysis. Surprisingly, ginsenoside RK1 exhibited negligible effects on the protein expression of LPCAT3 and ALOX12, pivotal enzymes in intracellular polyunsaturated fatty acid synthesis, as well as on GSS and GCL protein levels. However, high doses of ginsenoside RK1 were associated with decreased TFRC protein content and elevated FTH1 protein content. Furthermore, ginsenoside RK1 exerted a dose-dependent reduction in intracellular GCH1, DHODH, GPX4, and FSP1 protein levels, concomitant with an increase in intracellular PTGS2 protein levels (Figure 3A). Semi-quantitative assessment of Western blot grayscale values yielded consistent findings (Figure 3B–L). These observations suggest that ginsenoside RK1 predominantly induces ferroptosis in hepatocellular carcinoma by potentiating ferroptosis defense mechanisms.

### 2.4. Ginsenoside RK1 Promotes Ferroptosis in Hepatocellular Carcinoma by Down-Regulating FSP1

**Figure 4:**
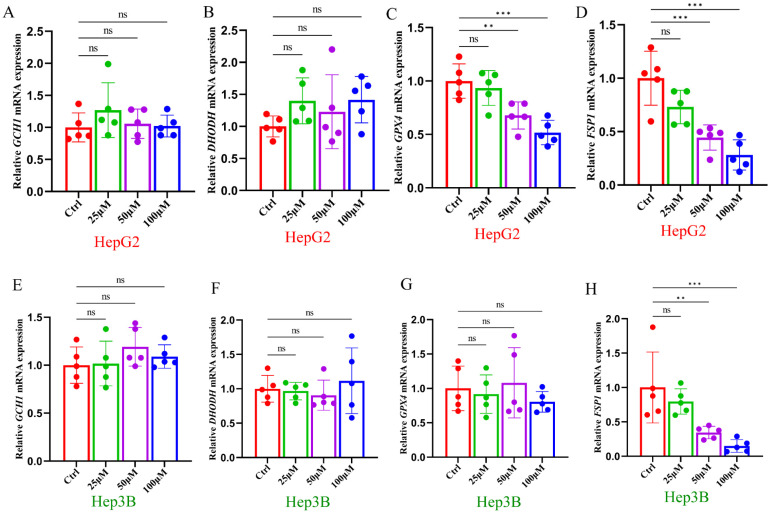
Ginsenoside RK1 induces ferroptosis by inhibiting FSP1 expression. (**A**–**D**) Gene express ion levels of *GCH1, DHODH, GPX4* and *FSP1* in HepG2 cells under the intervention of different concentrations of ginsenoside RK1 (*n* = 5). (**E**–**H**) Gene expression levels of *GCH1, DHODH, GPX4* and *FSP1* in Hep3B cells under the intervention of different concentrations of ginsenoside RK1 (*n* = 5). Data are mean ± SEM, *n* ≥ 3; data between two groups were analyzed by an independent samples *t*-test, and a one-way ANOVA was used to compare the means of three groups, ** *p* < 0.01, *** *p* < 0.001, ns: no significance.

To further elucidate the mechanism through which ginsenoside RK1 promotes ferroptosis by influencing key components of the ferroptosis defense axis, we assessed the mRNA expression levels of *GCH1*, *DHODH*, *GPX4*, and *FSP1* in HepG2 and Hep3B hepatocellular carcinoma cells following ginsenoside RK1 stimulation using RT-qPCR technology. Our analysis revealed a significant reduction in *GPX4* and *FSP1* mRNA levels in HepG2 cells upon ginsenoside treatment (Figure 4C,D), whereas *GCH1* and *DHODH* mRNA levels remained largely unaffected (Figure 4A,B). Similarly, in Hep3B cells, ginsenoside administration led to a decrease in *FSP1* mRNA levels (Figure 4H), while *GCH1*, *DHODH*, and *GPX4* mRNA levels remained unaltered (Figure 4E–G). Based on these findings, we postulate that *FSP1* likely plays a pivotal role in mediating ginsenoside RK1-induced ferroptosis in hepatocellular carcinoma cells.

### 2.5. Overexpression of FSP1 Alleviated Ginsenoside RK1-Induced Hepatocellular Carcinoma Cell Death

**Figure 5:**
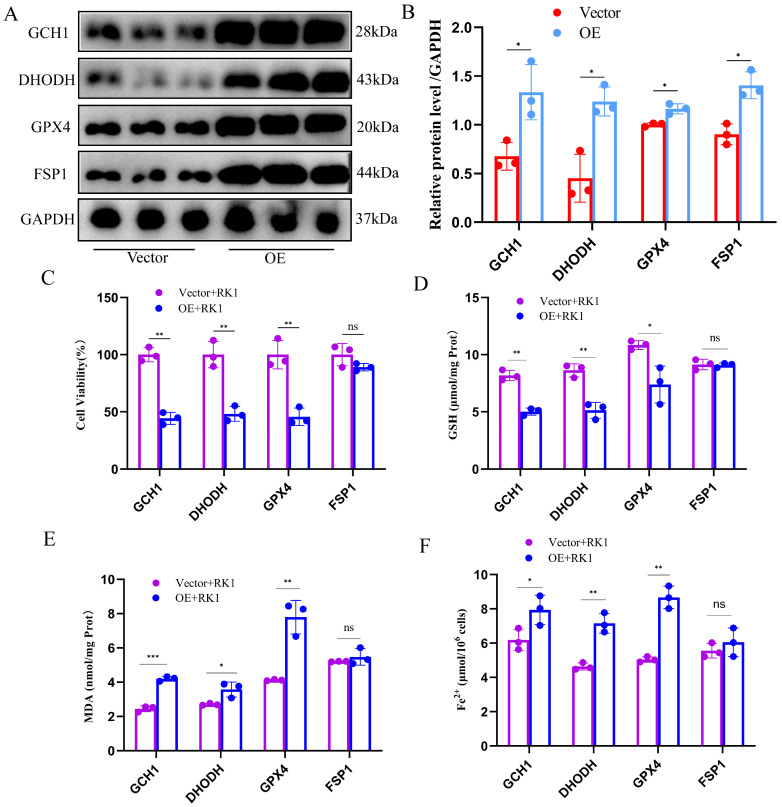
Overexpression of FSP1 inhibits ginsenoside RK1-induced ferroptosis in hepatocellular carcinoma cells. (**A**) Representative images of immunoblotting in HepG2 cells overexpressing *GCH1, DHODH, GPX4* and *FSP1*, respectively (*n* = 3). (**B**) Semi-quantitative statistical plots of overexpression of GCH1, DHODH, GPX4 and FSP1 in HepG2 cells, respectively (*n* = 3). (**C**) Effect of overexpression or non-expression of GCH1, DHODH, GPX4, and FSP1 on the viability of HepG2 cells under the intervention of ginsenoside RK1 (*n* = 3). (**D**) Effects of over- or under-expression of GCH1, DHODH, GPX4, and FSP1 on intracellular GSH content in HepG2 cells under the intervention of ginsenoside RK1 (*n* = 3). (**E**) Effects of over- or under-expression of GCH1, DHODH, GPX4, and FSP1 on intracellular MDA content in HepG2 cells under the intervention of ginsenoside RK1 (*n* = 3). (**F**) Effects of over- or under-expression of *GCH1, DHODH, GPX4*, and *FSP1* on intracellular iron ion content in HepG2 cells under the intervention of ginsenoside RK1 (*n* = 3). Data are mean ± SEM, *n* ≥ 3; data between two groups were analyzed by an independent samples *t*-test, and a one-way ANOVA was used to compare the means of three groups, * *p* < 0.05, ** *p* < 0.01, *** *p* < 0.001, ns: no significance.

To validate FSP1 as a central target of ginsenoside RK1-mediated ferroptosis in hepatocellular carcinoma, we engineered overexpression plasmids for *GCH1*, *DHODH*, *GPX4*, and *FSP1*, respectively, and transfected them into HepG2 cells. Western blot analysis confirmed the successful establishment of the overexpression system (Figure 5A,B). Subsequently, following the overexpression of the respective core proteins involved in ferroptosis defense, we assessed cell viability as well as levels of GSH, MDA, and iron ions in HepG2 cells. Remarkably, while overexpression of *GCH1*, *DHODH*, and *GPX4* failed to significantly increase cell viability in hepatocellular carcinoma cells, overexpression of FSP1 led to a substantial increase in cell viability (Figure 5C). Moreover, only *FSP1* overexpression notably counteracted the decrease in intracellular GSH levels induced by ginsenoside RK1 in HepG2 cells (Figure 5D). Similarly, FSP1 overexpression restored intracellular MDA levels to baseline levels and normalized intracellular iron ion levels in HepG2 cells (Figure 5E,F). Notably, overexpression of *GCH1*, *DHODH*, and *GPX4* did not mitigate the heightened ferroptosis sensitivity induced by ginsenoside RK1 in hepatocellular carcinoma cells. These results underscore FSP1 as a critical mediator of ginsenoside RK1-induced ferroptosis in hepatocellular carcinoma.

### 2.6. Silencing FSP1 Exacerbated Ginsenoside RK1-Induced Ferroptosis in Hepatocellular Carcinoma Cells

**Figure 6:**
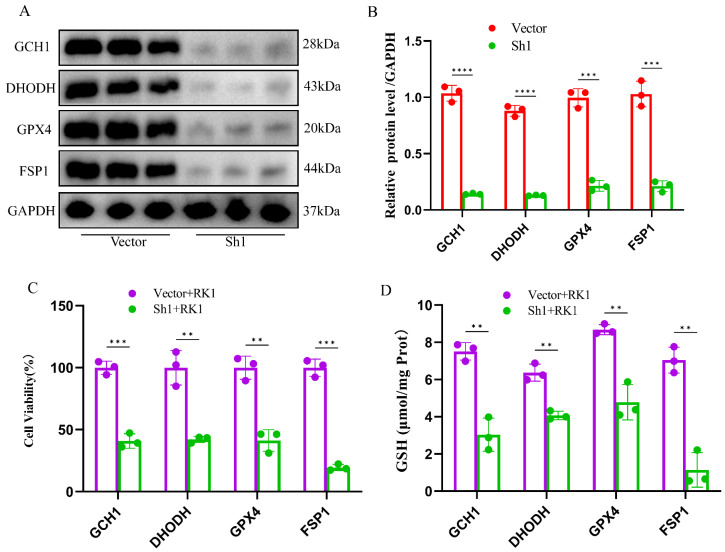

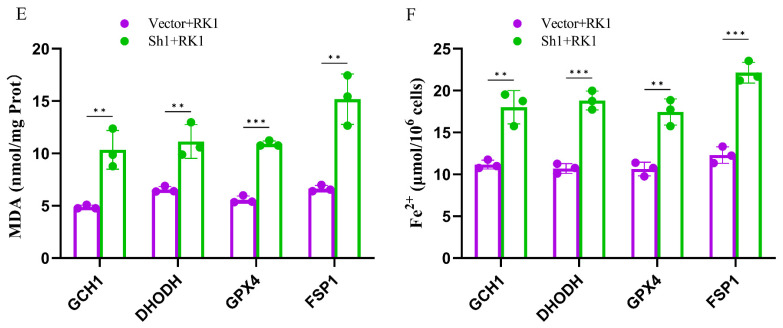
Knockdown of FSP1 promotes ginsenoside RK1-induced hepatocellular carcinoma. (**A**) Representative images of immunoblots knocking down GCH1, DHODH, GPX4 and FSP1 in HepG2 cells (*n* = 3). (**B**) Semi-quantitative statistics of knockdown of GCH1, DHODH, GPX4 and FSP1 in HepG2 cells (*n* = 3). (**C**) Effect of overexpression of GCH1, DHODH, GPX4 and FSP1 on ginsenoside RK1-mediated viability of HepG2 cells (*n* = 3). (**D**) Effect of overexpression of GCH1, DHODH, GPX4 and FSP1 on ginsenoside RK1-mediated GSH content in HepG2 cells (*n* = 3). (**E**) Effect of overexpression of GCH1, DHODH, GPX4 and FSP1 on ginsenoside RK1-mediated MDA content in HepG2 cells (*n* = 3). (**F**) Effect of overexpression of GCH1, DHODH, GPX4 and FSP1 on ginsenoside RK1-mediated iron ion content in HepG2 cells (*n* = 3). Data are mean ± SEM, *n* ≥ 3; data between two groups were analyzed by an independent samples *t*-test, and a one-way ANOVA was used to compare the means of three groups, ** *p* < 0.01, *** *p* < 0.001, **** *p* < 0.0001.

Furthermore, we generated knockdown plasmids targeting *GCH1*, *DHODH*, *GPX4*, and *FSP1*, which were subsequently transfected into HepG2 cells. Western blot analysis confirmed a significant suppression of *GCH1*, *DHODH*, *GPX4*, and *FSP1* expression levels (Figure 6A,B). Subsequent cell viability assays unveiled that silencing FSP1, followed by ginsenoside RK1 administration, failed to effectively curb the proliferation of HepG2 cells (Figure 6C). Notably, following FSP1 silencing, GSH levels in hepatocellular carcinoma cells rebounded (Figure 6D), while levels of MDA and iron ions decreased to baseline levels (Figure 6E,F). Conversely, knockdown of *GCH1*, *DHODH*, and *GPX4* did not elicit similar biological alterations. These findings underscore the pivotal role of *FSP1* in mediating ginsenoside RK1-induced ferroptosis in hepatocellular carcinoma cells.

## 3. Discussion

HCC stands out as one of the most prevalent malignant tumors globally, marked by its high rates of morbidity and mortality [19]. In contrast to the declining mortality trends observed in other common cancers like breast, lung, and prostate, HCC’s mortality rate persists in an upward trajectory, escalating by 2–3% annually [20]. Consequently, the quest for effective therapeutic targets and drugs represents a crucial avenue to circumvent the challenges encountered in hepatocellular carcinoma treatment [21,22]. Increasing evidence underscores the burgeoning significance of traditional Chinese medicine in targeting different genes to regulate ferroptosis bidirectionally, thus potentially offering novel therapeutic avenues for combating human diseases [23,24].

Since its inception, ferroptosis has been intimately intertwined with tumorigenesis. The discovery of erastin, a small molecule compound with selective cytotoxicity against KRAS-mutated tumor cells, unveiled ferroptosis as a form of iron-ion-dependent cell demise induced by lipid peroxide accumulation. Targeting ferroptosis not only directly inhibits tumor proliferation but also holds promise as a groundbreaking strategy to overcome tumor drug resistance and metastasis [15,25,26].

Intracellularly, the level of ferroptosis is intricately governed primarily by four major defense mechanisms within cells: (1) cystine/system Xc-/GSH/GPX4 [27]; (2) FSP1-CoQ_10_-NAD(P)H [28]; (3) DHODH-CoQ_10_-CoQ_H2_ [29,30]; and (4) GCH1/BH4/DHFR [31]. The GSH/GPX4 antioxidant system in the cytoplasm and mitochondria serves as one of the critical pathways for cells to resist ferroptosis. The cystine/glutamate antiporter (System Xc-) located on the cell membrane transports cystine into the cell and exports glutamate in a 1:1 ratio. Once cystine enters the cell, it is rapidly oxidized to cysteine, which is then used to synthesize GSH via glutamate-cysteine ligase (GCL) and glutathione synthetase (GSS). Utilizing GSH as a reducing cofactor, GPX4 in the cytoplasm and mitochondria can reduce lipid peroxides to lipid alcohols [15,32,33]. GCH1 and its metabolic derivatives, tetrahydrobiopterin and dihydrobiopterin, inhibit ferroptosis by selectively suppressing the consumption of phospholipids containing two polyunsaturated fatty acyl groups [34]. DHODH, a flavin-dependent enzyme located in the inner mitochondrial membrane, plays a crucial role in catalyzing the fourth step of the pyrimidine nucleotide synthesis pathway. This involves oxidizing dihydroorotic acid to orotic acid while transferring electrons to CoQ_10_ in the inner mitochondrial membrane, reducing it to CoQH2 [35]. This process aids in resisting ferroptosis within mitochondria. Additionally, studies have revealed that in cases of low GPX4 expression, DHODH can suppress lipid peroxidation and ferroptosis in mitochondria. However, when GPX4 is highly expressed, combining ferroptosis inducers with DHODH inhibitors significantly increases ferroptosis levels in tumor cells [36]. Impairment of any of these defense axes results in altered cellular susceptibility to ferroptosis. Notably, FSP1, also known as mitochondrial apoptosis-inducing factor 2, initially identified as a P53 response gene, is implicated in tumorigenesis and development regulation downstream of P53 [37]. Upon the addition of the ferroptosis inducer RSL3 to GPX4-deficient cells, sequencing analyses confirmed that FSP1 overexpression compensated for RSL3-induced ferroptosis post-GPX4 knockdown. Additional assays, including LDH release and cell activity assays, confirmed FSP1’s role in inhibiting ferroptosis independently of intracellular GSH levels, GPX4 activity, ACSL4 expression, or oxidizable fatty acid content [36]. Mechanistically, FSP1 catalyzes the reduction of CoQ_10_ to panthenol-10 at the plasma membrane, acting as a lipid-soluble antioxidant that mitigates lipid oxidative damage and ensuing ferroptosis [38]. The N-terminus of FSP1 bears a classical cardamoylation modification-related motif, influencing its interaction with the bilipid layer structure. Notably, mutation of this motif in the FSP1 (G2A) mutant abolished its anti-ferroptosis efficacy, underscoring the necessity of cardamoylation for FSP1 to exert its anti-ferroptosis effects [39].

The findings of this study demonstrate that ginsenoside RK1 selectively induces ferroptosis in hepatocellular carcinoma cells, significantly impeding their proliferation and viability. Concurrently, ginsenoside RK1 elevates intracellular lipid peroxide levels while reducing intracellular GSH levels and markedly increasing intracellular iron ion levels, thereby furnishing an ample substrate for intracellular ferroptosis initiation. The Fenton reaction triggered by iron ions serves as the source of intracellular lipid ROS. Our investigation elucidates that ginsenoside RK1 fosters ferroptosis in hepatocellular carcinoma cells by augmenting lipid peroxide generation while impeding their clearance. To delve into the specific mechanism underlying ginsenoside RK1-induced ferroptosis in hepatocellular carcinoma cells, we employed PCR and Western blotting techniques, revealing that ginsenoside RK1 heightens ferroptosis sensitivity primarily by diminishing the cells’ antioxidant capacity. Notably, FSP1 emerges as the pivotal target of ginsenoside RK1 in inducing ferroptosis in hepatocellular carcinoma cells, a conjecture substantiated by both overexpression and knockdown experiments. This could have significant implications for the therapy of solid tumors with high GPX4 expression in clinical settings. Typically, ferroptosis inducers have limited effectiveness against tumor types with high GPX4 expression. However, the mechanism by which ginsenosides induce ferroptosis does not rely on GPX4, providing a new strategy for targeting ferroptosis in patients with high GPX4 expression. Simultaneously, the combination of ginsenoside RK1 and FSP1 inhibition may produce a synergistic effect. This combination could reduce the activity of the anti-ferroptosis system within tumor cells, promoting the accumulation of lipid peroxides and further increasing the sensitivity of tumor cells to ferroptosis.

In summary, ginsenoside RK1 exerts antitumor effects by inducing ferroptosis in HepG2 and Hep3B cells. This mechanism is closely associated with the down-regulation of FSP1 enzyme activity by ginsenoside RK1, resulting in an oxidative–antioxidant system imbalance within hepatocellular carcinoma cells. This study provides a novel therapeutic approach for clinical treating HCC with ginsenoside RK1 and establishes a scientific rationale for its utilization in patient care. However, the extent to which ginsenoside RK1 induces ferroptosis, the varying resistance of different programmed cell death modes across tumors, and the crosstalk between different types of cell death remain pressing issues to be addressed.

## 4. Materials and Methods

### 4.1. Cells

Hepatocellular carcinoma HepG2 and Hep3B cell lines were purchased from the cell bank of Chinese Academy of Sciences. Cells were authenticated using STR profiling. 

### 4.2. Reagents and Antibodies

Ginsenoside RK1 (Figure S1), purity: 99.64% (HY-N2515), Ferrostatin-1 (Fer-1) (HY-100579), and Liprostatin-1 (Lip-1) (HY-12726) were purchased from MedChem Express (Monmouth Junction, NJ, USA). A glutathione (GSH) kit (S0052) and malondialdehyde (MDA) detection kit were purchased from Shanghai Beyotime Biotechnology Co. (S0131M, Shanghai, China). A Cell Proliferation-Toxicity Assay CCK-8 Kit (CX001), Universal Antibody Diluent (PS119L), and Protein-Free Rapid Sequester were purchased from Shanghai Yase Biomedical Technology Co. (PS108P, Shanghai, China). Ferroptosis Suppressor Protein 1 (FSP1) antibody (1:5000, A22278), Glutathione Peroxidase 4 (GPX4) antibody (1:1000, A11243), GTP Cyclization Hydrolyzing Enzyme 1 (GCH1) antibody (1:1000, A10616), and GAPDH antibody (1:10,000, A19056) were purchased from ABclonal Technology (Wuhan, China). An iron ion detection kit (G4301) and dihydroorotic acid dehydrogenase (DHODH) antibody (1:500) were purchased from Wuhan servicebio Biotechnology Co., Ltd. (GB112641, Wuhan, China). An RNA reverse transcription kit (R223-01) and RNA amplification kit were purchased from Nanjing Vazyme Biotech Co., Ltd. (Q711-02, Nanjing, China). Transfection reagents were purchased from Polyplus, Illkirch-Graffenstaden, France. Primers were synthesized by Shanghai Sangong Bioengineering Co., Ltd. (Shanghai, China), and the primer sequences are shown in Table 1. *FSP1, GCH1, DHODH* and *GPX4* overexpression and knockdown plasmids were designed and synthesized by Shanghai Genechem Co., Ltd. (Shanghai, China).

### 4.3. Main Instruments

An ABI7500 real-time quantitative PCR instrument was purchased from Applied Bios systems (Waltham, MA, USA); a FACScan flow cytometer was purchased from BectonDickinson (Franklin Lakes, NJ, USA); a 5804R high-speed cryo-centrifuge was purchased from Eppendorf (Leipzig, Germany); an EXL800 enzyme labeling instrument was purchased from BioTek (Winooski, VT, USA); and a DMi8 fluorescence microscope was purchased from BioTek (Bad Friedrichshall, Germany). A fluorescence microscope was purchased from Leica,(Wetzlar, Germany).

### 4.4. Cell Culture

HepG2 and Hep3B cells were cultured in DMEM culture medium supplemented with 10% fetal bovine serum (FBS) and 1% penicillin and streptomycin, and the cells were incubated at 37 °C in an incubator maintained in a logarithmic growth state in a 5% CO_2_ environment.

### 4.5. CCK8 Experiment

Cells were seeded at 3000 per well in 96-well plates, and after the cells were attached to the wall, 10 μM, 20 μM and 40 μM of ginsenoside RK1 was added according to the corresponding groups to stimulate the cells for 24 h. After removing the supernatant, 100 μL of medium containing 10% CCK8 solution was added to each well, and the absorbance was detected under the wavelength of 450 nm of the enzyme counter for a total of 1 h. The cells were incubated for 1 h with a total of 1.4 μm of ginseng saponin in the medium containing CCK8, and the absorbance was measured at the wavelength of 450 nm. Relative cell viability (%) = (measured value − blank value)/(control value − blank value) × 100%.

### 4.6. GSH, Malondialdehyde (MDA) Assay

Cells were seeded at 8000 per well in a six-well plate, and after the cells were attached to the wall for 24 h, 10 μM, 20 μM and 40 μM of ginsenoside RK1 was given to stimulate the cells for 24 h; afterwards, the cells were lysed and centrifuged at 12,000 rpm for 10 min, and then the supernatant was taken and detected according to the operation of the kit.

### 4.7. Western Blot Detection of Corresponding Protein Expression Levels

Cells were seeded at 8000 per well in six-well plates, and after the cells were attached to the wall, the cells were stimulated with 10 μM, 20 μM and 40 μM ginsenoside RK1 for 24 h, the supernatant was aspirated and discarded, the cells were washed three times with PBS, and the cells were lysed in RIPA lysing solution. Protease inhibitor and phosphatase inhibitor were added, the cells were lysed on ice, and then the cells were centrifuged at 12,000 rpm for 10 min. Then, the supernatant was taken for the determination of the concentration of BCA proteins, and the equivalent amount of protein (30 μg) was detected. An equal amount of protein (30 μg) was separated by vertical electrophoresis on 10% SDS-PAGE and then transferred to the PVDF membrane. The PVDF membrane was incubated in protein-free rapid containment solution for 15 min, and then FSP1 (1:5000), GPX4, DHODH, and GCH1 were added, respectively, at 4° overnight. The next day, HRP-labeled secondary antibody was added, and chemiluminescence with ECL chromogen. The expression level of target proteins was analyzed semi-quantitatively via Image J (1.8.0, NIH, USA) using GAPDH as internal reference.

### 4.8. RT-qPCR Detection of Corresponding mRNA Expression Level

Total cellular RNA was extracted and reverse-transcribed into cDNA. cDNA was mixed with primers and Universal SYBR qPCR Master Mix at 95 °C for 2 min, 95 °C for 10 s, 60 °C for 30 s, and 40 cycles for amplification. cDNA was read as a CT value for subsequent statistical analysis.

### 4.9. Plasmid Transfection

Polyplus reagent, pCMV 3 and empty pCMV3 vector were applied to transfect HepG2 cells. HepG2 cells were inoculated in 6-well plates at a density of 1 × 10^6^ cells per well. After 24 h of cell growth, 2 μg of empty, overexpressed or knockdown plasmid, 4 μL of transfection reagent, 200 μL of buffer, and 2 mL of serum-free medium were added to each well, the complete medium was replaced after 6 h of transfection, and the complete medium was changed with or without the addition of ginsenoside RK1 (20 μM) according to different groups for subsequent experiments. The specific groups included an empty + ginsenoside RK1 group, an overexpression group, and a knockdown group + ginsenoside RK1 group.

### 4.10. Statistical Methods

The data were statistically analyzed with GraphPad Prism 9 software, and the measurement data between two groups conformed to normal distribution using both the Chi-square and *t*-test. Comparisons between multiple groups were analyzed using a one-way ANOVA, and further comparisons between multiple groups were made via Tukey’s test. The Kruskal–Wallis H test was used if the data did not conform to normal distribution. All data are expressed as $(\bar{X}\pm S$), and differences are considered statistically significant at *p* < 0.05.

## Figures and Tables

**Table 1:** List of Primer Sequences for Each Gene.

Gene Name	Primer Sequence (5′~3′)	Product Length/bp
*FSP1*	F:GCGTGCCCTCTGGAGAAG R:TCGTCGGGTCCTTCTTTACT	303
*GCH1*	F:CGAGCTGAACCTCCCTAACC R:CACACCCAACATTGTGCTGG	508
*DHODH*	F:GGAAGTGAGAGTTCTGGGCC R:CACACTGGCAATGTCCTCCT	577
*GPX4*	F:GCCAGGGAGTAACGAAGAGA R:CAGCCGTTCTTGTCGATGAG	198
*GAPDH*	F:CCAGAACATCATCCCTGCCT R:CCTGCTTCACCACCTTCTTG	185

## Data Availability

Data are contained within the article.

## Supplementary Materials

The following supporting information can be downloaded at: https://www.mdpi.com/article/10.3390/ph17070871/s1, Figure S1: Structural formula of ginsenoside RK1; Figure S2: Effects of ginsenoside RK1 on hepatocyte toxicity and FSP1 expression.
