# Supplementary material for: Ginsenoside RK1 Induces Ferroptosis in Hepatocellular Carcinoma Cells through an FSP1-Dependent Pathway
Source: Pharmaceuticals (Basel). 2024 Jul 2;17(7):871. doi: 10.3390/ph17070871 (PMC11279434; doi:10.3390/ph17070871)
Supplement: Supplementary file 1 [file pharmaceuticals-17-00871-s001.zip › Figure S2.pdf]

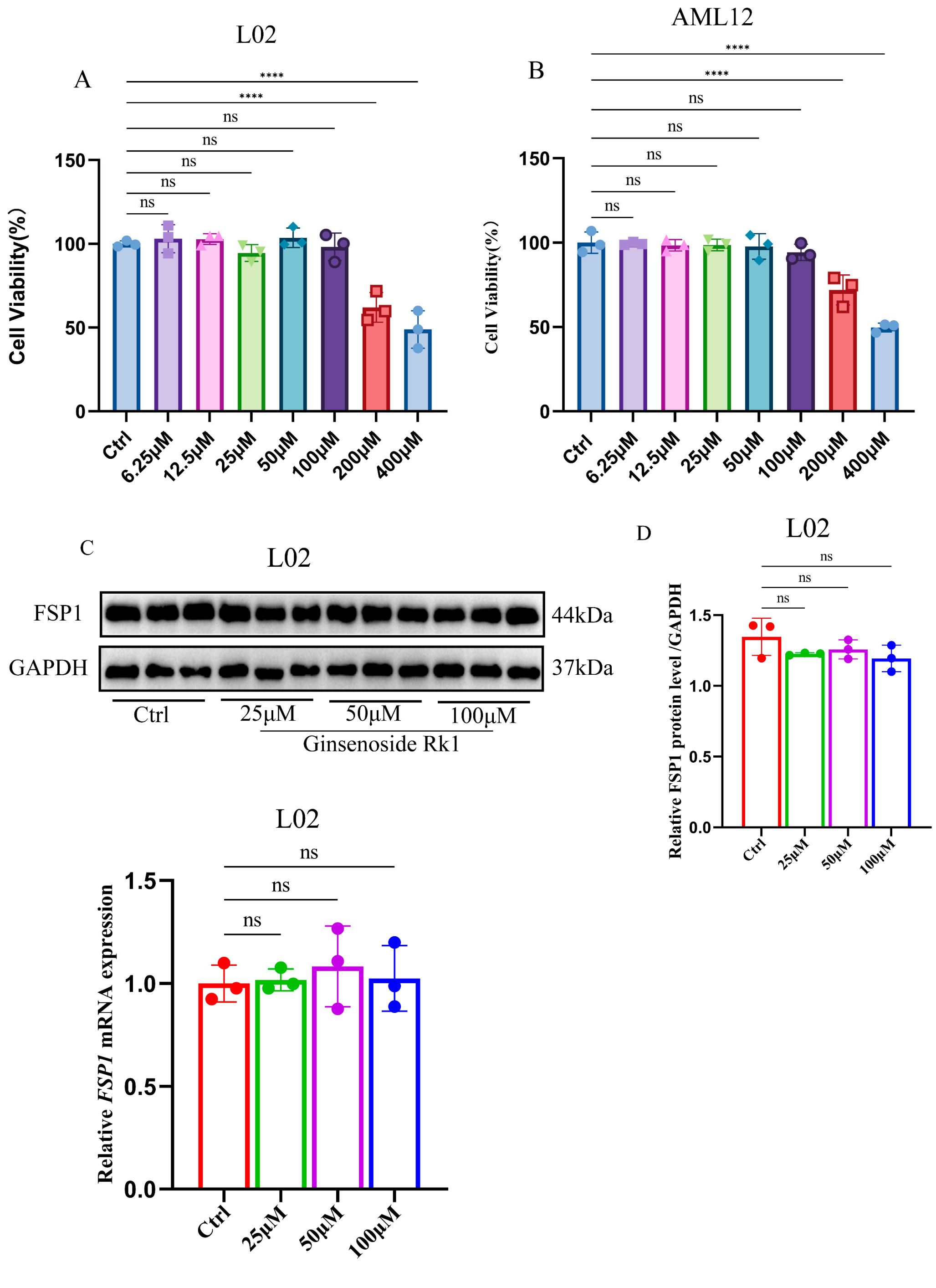

Figure S2. Effects of ginsenoside RK1 on hepatocyte toxicity and FSP1 expression.

(A) Effects of different concentrations of curcumin on L02 viability of human hepatocytes. (B) Effect of ginsenoside RK1 on AML12 cell viability in mouse hepatocytes. (D-E) Immunoblot and semi-quantitative statistics of FSP1 protein content in L02 cells under ginsenoside RK1 stimulation. (F) Effect of FSP1 gene in L02 cells under ginsenoside RK1 stimulation. ****$P < 0.0001$, ns: no significance.
