# Supplementary figures and images for: Ginsenoside RK1 Induces Ferroptosis in Hepatocellular Carcinoma Cells through an FSP1-Dependent Pathway
Source: Pharmaceuticals (Basel). 2024 Jul 2;17(7):871. doi: 10.3390/ph17070871 (PMC11279434; doi:10.3390/ph17070871)

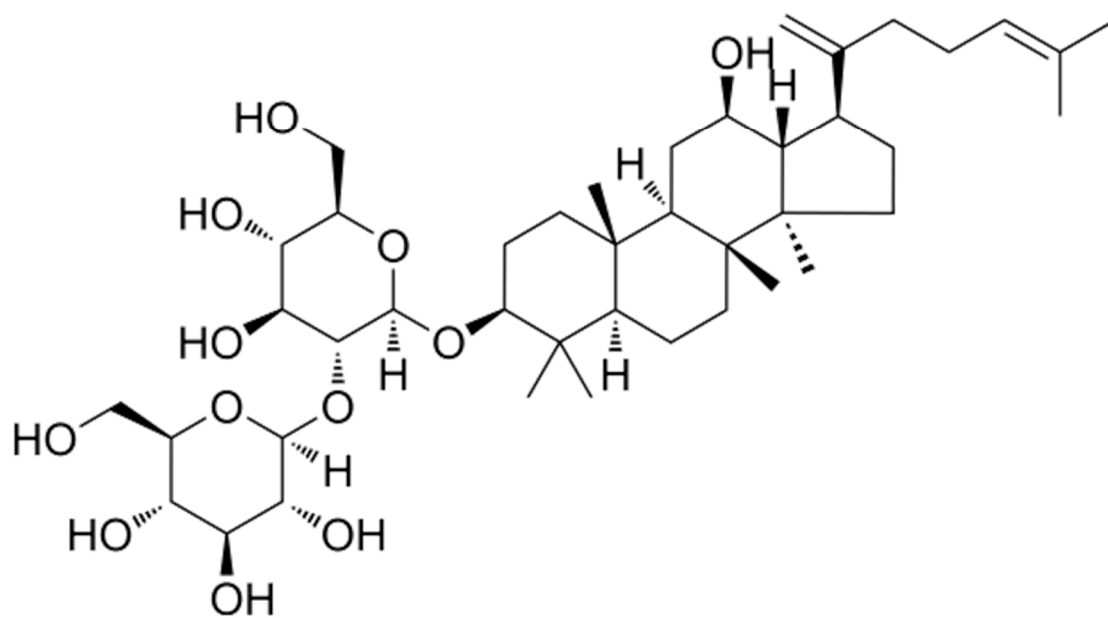

**Figure S1. Structural formula of ginsenoside RK1**

Supplement: Supplementary file 1 [file pharmaceuticals-17-00871-s001.zip › Figure S1.pdf]
